# Supplementary material for: THE INFLUENCE OF MUSCLE MASS ON THE COORDINATION REQUIRED FOR EFFICIENT MOVEMENT
Source: bioRxiv. 2026 May 5:2026.04.30.722018. Preprint. [Version 2] doi: 10.64898/2026.04.30.722018 (PMC13174556; doi:10.64898/2026.04.30.722018)
Supplement: Supplement 1 [file media-1.pdf]

The influence of muscle mass on the coordination required for efficient movement

Table S1. Characteristics of the 12 participants.

Label	Gender	Age (years)	Body mass (kg)	Height (m)
S02	male	28	68.0	1.67
S03	female	30	65.5	1.67
S04	male	33	68.0	1.75
S05	female	43	64.4	1.74
S06	male	32	63.0	1.83
S07	male	37	71.8	1.73
S11	male	31	82.8	1.83
S12	female	24	65.8	1.71
S13	female	32	63.5	1.68
S14	male	24	78.9	1.78
S15	female	26	58.0	1.67
S17	female	26	80.7	1.73

Table S2. Statistical results for the GLM ANOVA for the PC loading scores for the first 25 PCs. Significants (p) is displayed for both the muscle-mass scale (Mass) and the pedal condition (Cond).

PC	$F_{\text{Mass}}(4, 645)$	p_{Mass}	$F_{\text{Cond}}(3, 645)$	p_{Cond}
1	0.25	$p \geq 0.05$	350.50	$p < 0.001$
2	20.58	$p < 0.001$	71.96	$p < 0.001$
3	0.69	$p \geq 0.05$	15.18	p<0.001
4	49.09	$p < 0.001$	32.63	$p < 0.001$
5	79.82	$p < 0.001$	9.94	$p < 0.001$
6	139.22	$p < 0.001$	40.79	$p < 0.001$
7	11.61	$p < 0.001$	44.76	$p < 0.001$
8	29.45	$p < 0.001$	11.76	$p < 0.001$
9	14.78	$p < 0.001$	59.64	$p < 0.001$
10	4.69	$p < 0.001$	11.08	$p < 0.001$
11	5.70	$p < 0.001$	7.90	$p < 0.001$
12	21.65	$p < 0.001$	13.55	$p < 0.001$
13	0.76	$p \geq 0.05$	18.43	$p < 0.001$
14	4.92	$p < 0.001$	22.01	$p < 0.001$
15	0.50	$p \geq 0.05$	8.94	$p < 0.001$
16	0.063	$p \geq 0.05$	15.62	$p < 0.001$
17	0.84	$p \geq 0.05$	19.42	$p < 0.001$
18	0.74	$p \geq 0.05$	31.30	$p < 0.001$
19	2.19	$p \geq 0.05$	21.92	$p < 0.001$
20	0.13	$p \geq 0.05$	7.07	$p < 0.001$
21	0.17	$p \geq 0.05$	4.50	$p < 0.05$
22	0.42	$p \geq 0.05$	6.14	$p < 0.001$
23	0.31	$p \geq 0.05$	7.89	$p < 0.001$
24	0.22	$p \geq 0.05$	8.50	$p < 0.001$
25	0.06	$p \geq 0.05$	2.4	$p \geq 0.05$

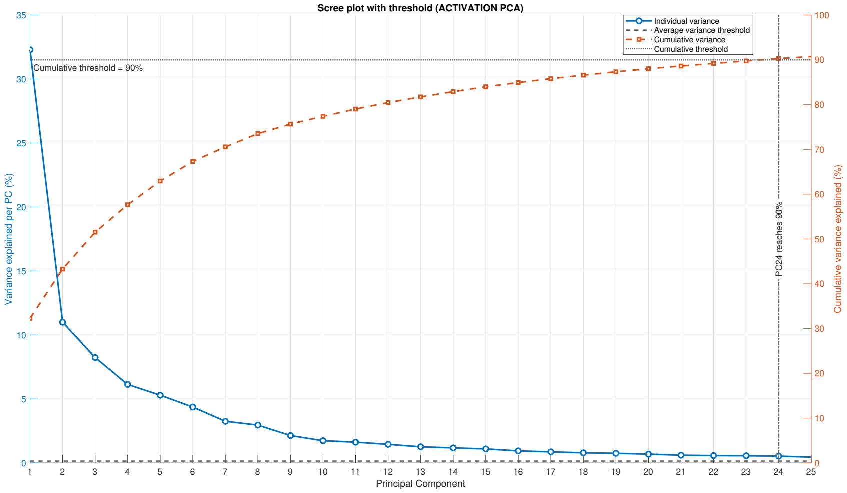

Fig. S1. Scree plot showing the percentage of the variance of the muscle coordination patterns explained by the first 25 PCs.

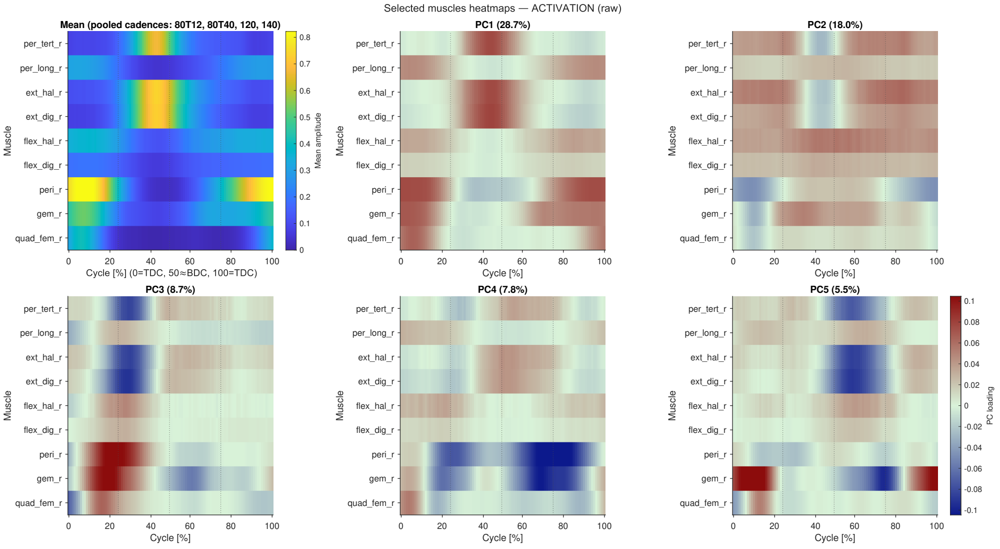

Fig. S2. The mean activation pattern and the weights for the first five Principal Components (PCs: 1-5). The variance explained is shown for each PC. Time is normalised to a percentage of the pedal cycle, starting and finishing with the crank at top-dead-centre.

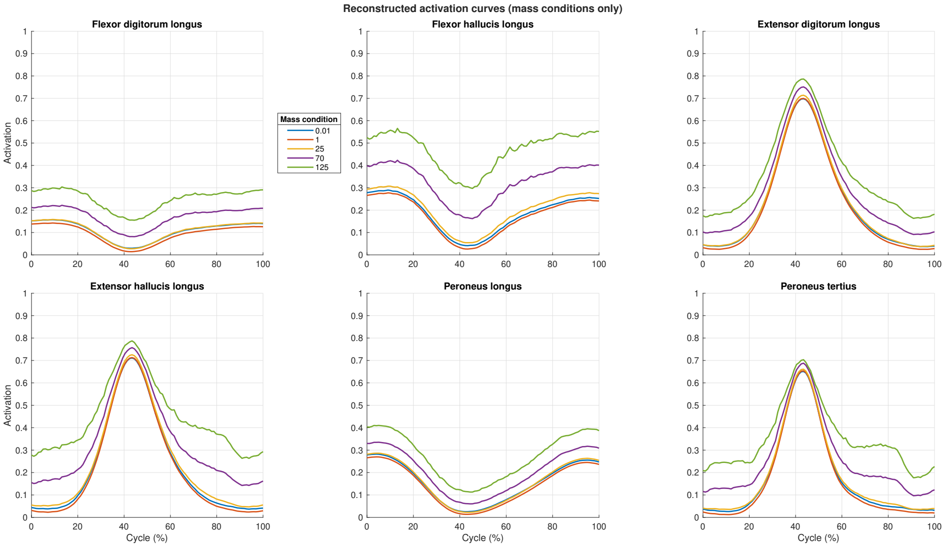

Fig. S3. Reconstructed activation patterns for the other muscles using the mean activation and vector product of the loading scores and weights for PCs1-14. The statistical effect of the muscle-mass scale is shown. Loading scores were only selected to differ between conditions if there was a significant effect of scale for that PC from the ANOVA.
